# Successful Treatment of Unresectable Advanced Melanoma by Administration of Nivolumab With Ipilimumab Before Primary Tumor Resection

**DOI:** 10.3389/fmed.2019.00140

**Published:** 2019-06-26

**Authors:** Taku Fujimura, Yumi Kambayashi, Yota Sato, Kayo Tanita, Ryo Amagai, Akira Hashimoto, Takanori Hidaka, Setsuya Aiba

**Affiliations:** Department of Dermatology, Tohoku University Graduate School of Medicine, Sendai, Japan

**Keywords:** nivolumab, ipilimumab, advanced melanoma, pre-surgical administration, T cell expansion

## Abstract

Ipilimumab, in combination with nivolumab, is one of the promising drugs that enhance the anti-tumor immune response of patients with advanced melanoma. Since the co-administration of nivolumab with ipilimumab in the neoadjuvant setting expands melanoma-reactive T cells at the primary site of melanoma and has a high rate of histological complete response, the pre-surgical administration of this combination could be the optimal therapy for unresectable advanced melanoma. In this report, a case of unresectable advanced melanoma treated successfully with administration of nivolumab with ipilimumab before primary tumor resection is presented. In addition, CD8+ T cells increased among the tumor-infiltrating lymphocytes that were surrounding melanoma cells and caspase 3+ cells. The present case suggests that pre-surgical administration of nivolumab with ipilimumab could be the optimal therapy for the treatment of unresectable advanced melanoma.

## Background

Nivolumab, anti-PD1 antibody (Abs), monotherapy has been one of the first-line therapies for advanced melanoma, especially for BRAF mutation-negative melanoma, with a reported efficacy rate of ~30–40% (1, 2). Ipilimumab, cytotoxic T-lymphocyte antigen (CTLA-4) Abs, is another immune checkpoint inhibitor (ICI) for the treatment of advanced melanoma that activates and increases T cells, and it expands effector T cells at the site especially when given with nivolumab (3). Therefore, pre-surgical treatment with the combination of nivolumab and ipilimumab could be optimal therapy for unresectable, advanced melanoma.

## Case Presentation

**Figure 1:**
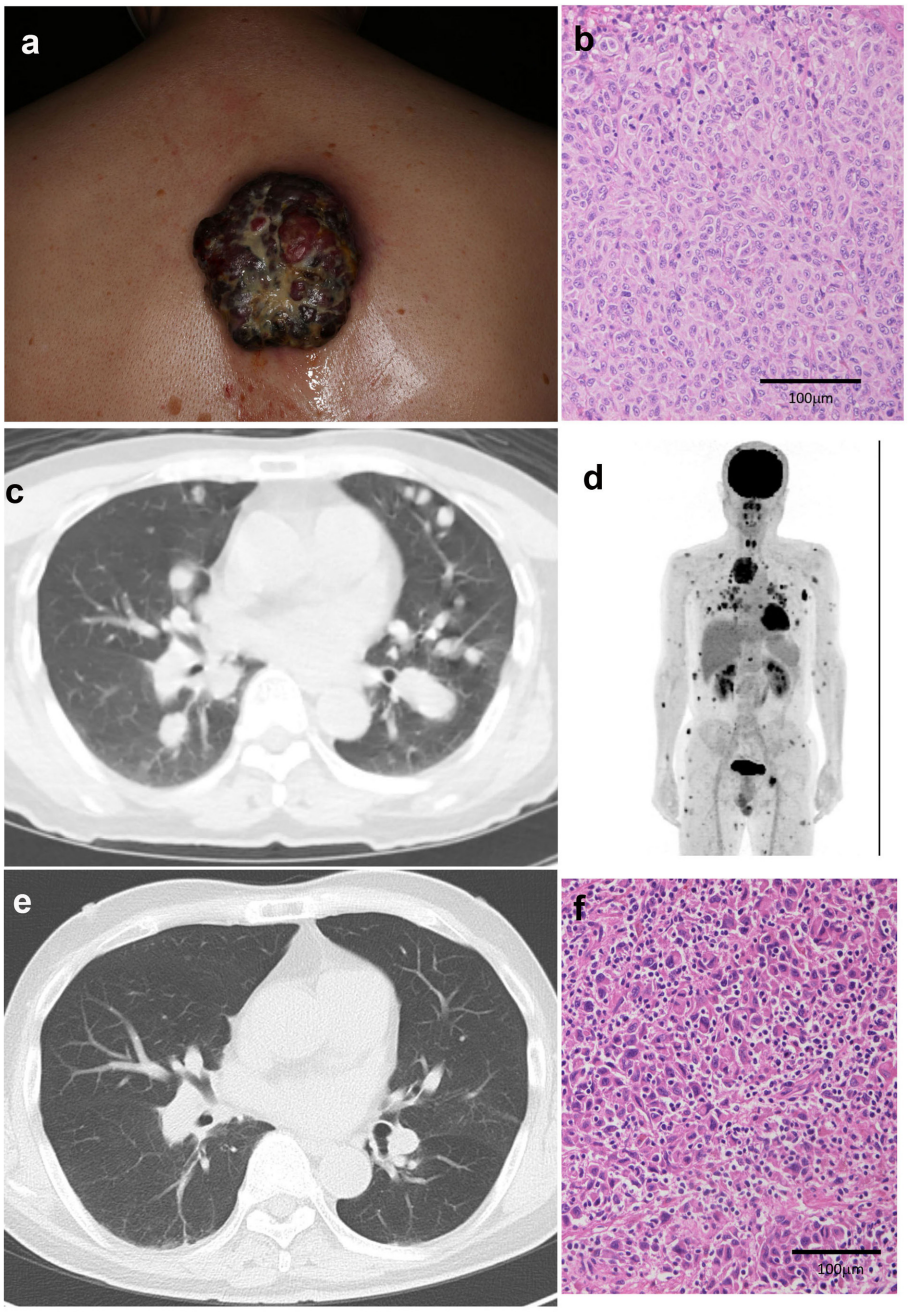
A black nodule (8 × 7 cm) with a dark-red nodule on the back **(a)**. Histological findings of the primary tumor before treatment: markedly atypical melanocytes arranged in irregular nests and solitary units **(b)**. Multiple lung metastases **(c)**, cutaneous, pharyngeal, and peritoneal nodules, lymph node metastases, and bone metastases on PET-CT **(d)**. After treatment, multiple lung metastases are decreased **(e)**. Histological findings of the primary tumor after single administration of nivolumab with ipilimumab showing dense infiltration of lymphocytes in the melanoma lesion **(f)**.

A 64-year-old Japanese man visited our outpatient clinic with a 3-months history of an easily bleeding, black nodule on his back. At the initial physical examination, a black nodule (8 × 7 cm) with a dark-red nodule was seen on the back (Figure 1a). In addition, there were numerous subcutaneous nodules on the scalp, face, trunk, and extremities. Biopsy of the primary tumor showed markedly atypical melanocytes arranged in irregular nests and solitary units (Figure 1b). The THxID kit revealed that the primary tumor possessed the BRAF^V600E^ mutation. Immunohistochemical staining showed that these melanoma cells were positive for Melan A and HMB45. PET-CT showed multiple lung (Figure 1c), cutaneous, pharyngeal, and peritoneal nodules, as well as lymph node and bone metastases (Figure 1d). Biopsy from the pharyngeal wall showed dense infiltration of markedly atypical melanocytes. In addition, serum LDH levels were elevated (336 U/l). From the above findings, the diagnosis was malignant melanoma with multiple lung, peritoneal, pharyngeal, subcutaneous, lymph node, and bone metastases [pT4bN3cM1c(1) stage IV].

## Treatment Course and Outcome

Since the patient had metastases in 6 organs (>3 organs) and elevated serum LDH levels, suggesting that dabrafenib plus trametinib combined therapy might not be useful (4), nivolumab (80 mg/body/every 3 weeks) was given in combination with ipilimumab (3 mg/kg/every 3 weeks) before surgical treatment. Eighteen days after the administration of nivolumab and ipilimumab, the primary tumor was palliatively resected, and nivolumab (80 mg/body/every 3 weeks) in combination with ipilimumab (3 mg/kg/every 3 weeks) was continued for three more cycles (Supplemental Figure 1). The skin metastases regressed rapidly with scar formation, and follow-up CT 2 months after the combination therapy suggested significant regression of lung (Figure 1e), peritoneal, pharyngeal, subcutaneous, lymph node, and bone metastases. Histological findings of the resected primary tumor showed dense infiltration of lymphocytes in the melanoma lesion (Figure 1f). Four months have passed, and a grade 3 skin rash and grade 4 peripheral neuropathy, which is controlled by the intravenous administration of methylprednisolone sodium succinate at a starting dose of 2 mg/kg, were observed.

## Immunohistochemical Investigation of Tumor Infiltrating Lymphocytes (TILs)

**Figure 2:**
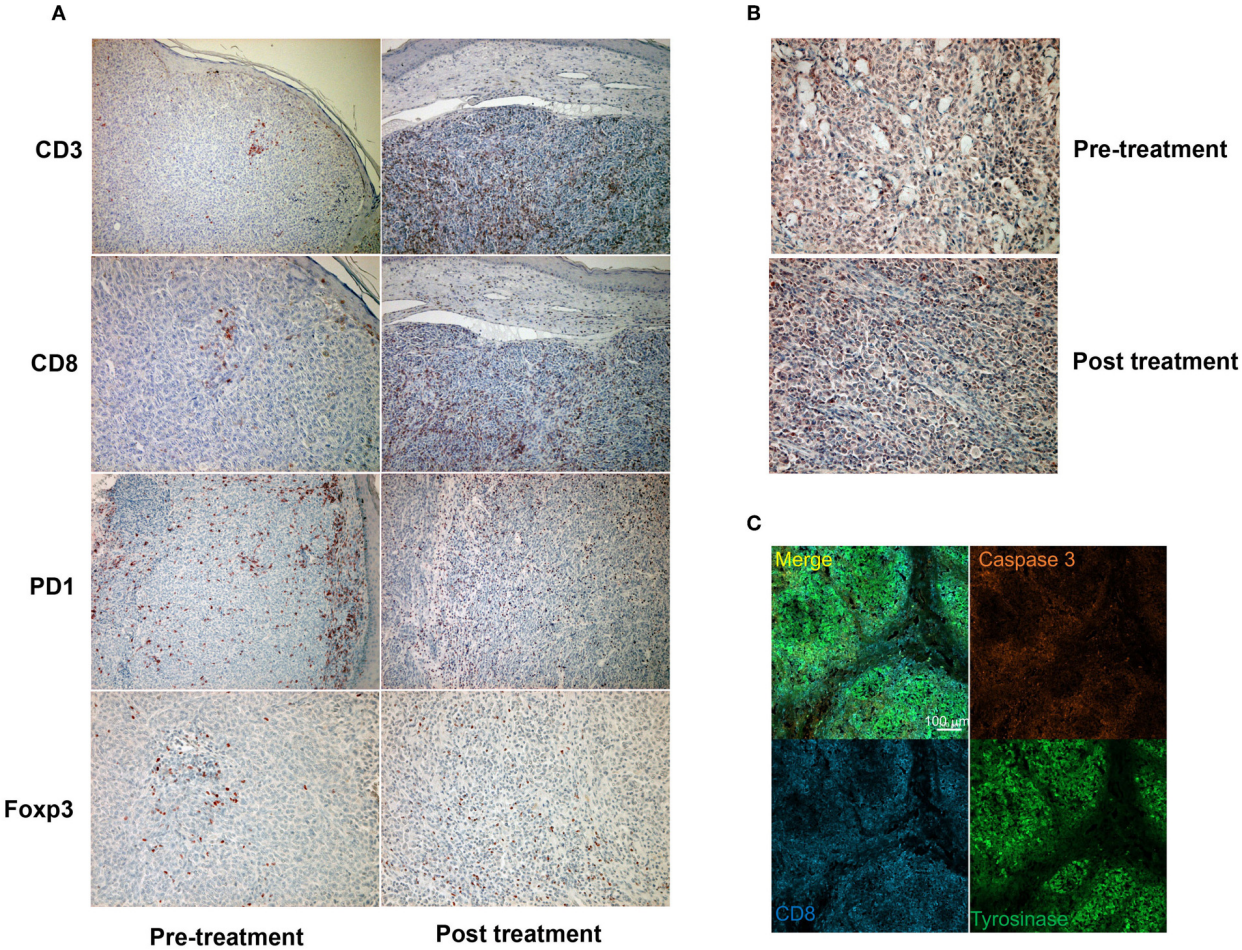
Immunohistochemical staining for CD3, CD8, PD1, and Foxp3 before and after a single administration of nivolumab with ipilimumab **(A)**. Immunohistochemical staining for PD-L1 before and after a single administration of nivolumab with ipilimumab **(B)**. Immunofluorescence staining of CD8 (cytotoxic T cells: blue), caspase 3 (apoptotic cells: orange), and tyrosinase (melanoma cells: green) **(C)**.

**Figure 3:**
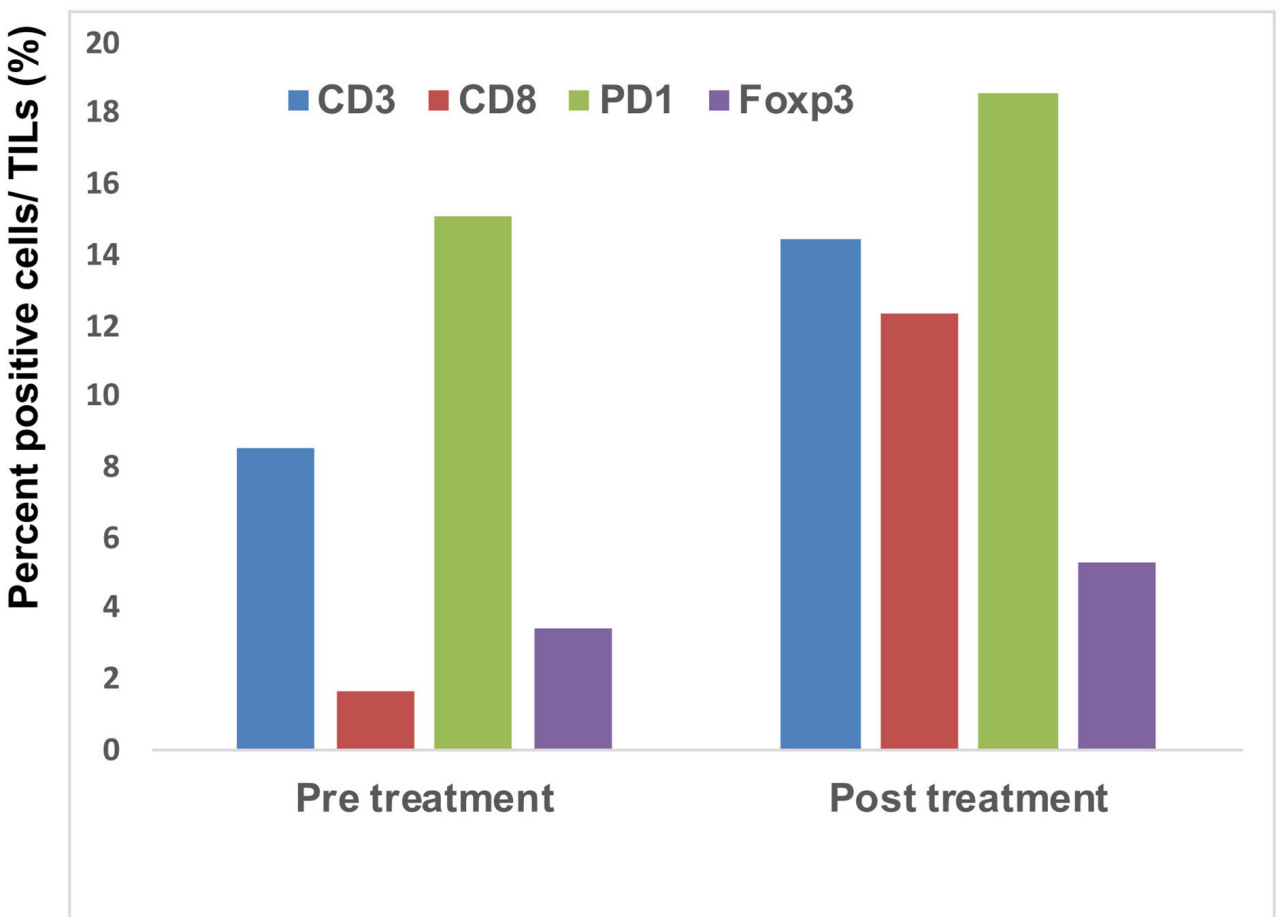
Quantitative analysis of CD3+ T cells, CD8+ T cells, PD1-expressing cells, and Foxp3+ cells: the IHC-positive cells within the lymphocyte fraction and the percentage of IHC-positive cells per all tumor-infiltrating cells were automatically counted using a BZ-X800.

Since a previous study suggested that combination therapy with nivolumab and ipilimumab significantly increased a neoantigen-specific melanoma-resident T cell clone, inducing a durable anti-immune response in melanoma patients (1), immunohistochemical staining for CD3 and CD8 was performed before and after the administration of combination therapy (Figure 2A). The ratios of CD3, CD8, PD1, and Foxp3+ cells among tumor infiltrating lymphocytes (TILs) in the primary tumor before the administration of nivolumab plus ipilimumab combination therapy and in the primary tumor 18 days after the administration of combined therapy were analyzed using the BZ-X800 (KEYENCE, Tokyo, Japan). The lymphocyte fractions, CD3+ cells, CD8+ cells, PD1+ cells, and Foxp3+ cells, were counted, and the ratios of cells staining positive on immunohistochemistry (CD3+ cells/total TILs, CD8+ cells/total TILs, PD1+ cells/total TILs, Foxp3+ cells/total TILs) were calculated in the full tumor areas of low magnification fields. These data showed a marked increase of CD8+ TILs in the post-treatment specimen (Figure 3). In addition, immunohistochemical staining for PD-L1 was performed, showing no difference between before and after the administration of combination therapy (Figure 2B). Moreover, immunofluorescence staining for caspase 3, CD8, and tyrosinase showed the induction of apoptotic cells in the melanoma lesion (Figure 2C).

### Ethics Statement

The patient gave written informed consent for the publication of this case report.

## Discussion

Ipilimumab, in combination with nivolumab, is one of the promising drugs that enhance the anti-immune response of patients with advanced melanoma with or without BRAF gene mutation (5–7). Indeed, the response rate to this combination therapy for advanced melanoma has been reported to be 57.8% (7), and it is recommended by the NCCN guideline for cutaneous melanoma as a first-line therapy (8), despite its high toxicity. In addition, this combination therapy has a high efficacy rate even for the treatment of brain metastases of melanoma (5). Notably, Blank et al. reported that the efficacy of the combination of nivolumab and ipilimumab in the neoadjuvant setting does not parallel tumor mutation burden (TMB) and achieves a high histological complete response (CR) rate (3), suggesting that pre-surgical administration of nivolumab with ipilimumab could be the optimal treatment for unresectable advanced melanoma in real-world practice. In addition, the efficacy of a BRAF inhibitor combo, such as dabrafenib and trametinib combination therapy or encorafenib and binimetinib combination therapy, is limited in advanced melanoma with multiple organ metastases (4, 9). Although there is still insufficient evidence for the efficacy of pre-operative treatment by nivolumab plus ipilimumab, these reports suggested that pre-operative treatment by nivolumab plus ipilimumab might induce a stronger and broader tumor-specific T cell response, as in a pre-clinical study (10). In addition, Navarrete-Dechent reported one case of stage III, unresectable melanoma treated with nivolumab plus ipilimumab combined therapy, and evaluated its efficacy using reflectance confocal microscopy (11). Their report suggested that ipilimumab plus nivolumab combined immune therapy is useful for the treatment of unresectable melanoma (11).

Concerning the present case, although the patient had at least 6 organ metastases, pre-surgical administration of nivolumab with ipilimumab dramatically reduced tumor masses in all organs. Interestingly, a single administration of this combination therapy increased the ratio of CD8+ T cells among total TILs from 1.7 to 12.3% (Figure 3). Moreover, immunofluorescence staining showed that caspase 3+ apoptotic cells were surrounded by CD8+ T cells in the melanoma area, suggesting that increased CD8+ T cells might directly induce apoptosis of melanoma cells. Taken together, administration of nivolumab with ipilimumab before primary tumor resection increased CD8+ T cells in the primary tumor, probably a melanoma-specific T cell clone, inducing a systemic anti-melanoma immune response in advanced melanoma. To prove this hypothesis, another clinical study to evaluate nivolumab plus ipilimumab combination therapy prior to surgery is needed.

## Data Availability

All datasets for this study are included in the manuscript and/or the Supplementary Files.

## Ethics Statement

This patient gave written informed consent.

## Author Contributions

TF designed the research study and wrote the manuscript. TF, YK, YS, KT, and TH performed and analyze the IHC staining. TF, YK, RA, and AH treated the patients and acquired the clinical data and samples. TF and SA supervised the study.

### Conflict of Interest Statement

The authors declare that the research was conducted in the absence of any commercial or financial relationships that could be construed as a potential conflict of interest.

## Supplementary Material

Supplemental Figure 1 Primary tumor on the back before and after the administration of nivolumab plus ipilimumab.
