# Supplementary figures and images for: Successful Treatment of Unresectable Advanced Melanoma by Administration of Nivolumab With Ipilimumab Before Primary Tumor Resection
Source: Front Med (Lausanne). 2019 Jun 26;6:140. doi: 10.3389/fmed.2019.00140 (PMC6607934; doi:10.3389/fmed.2019.00140)

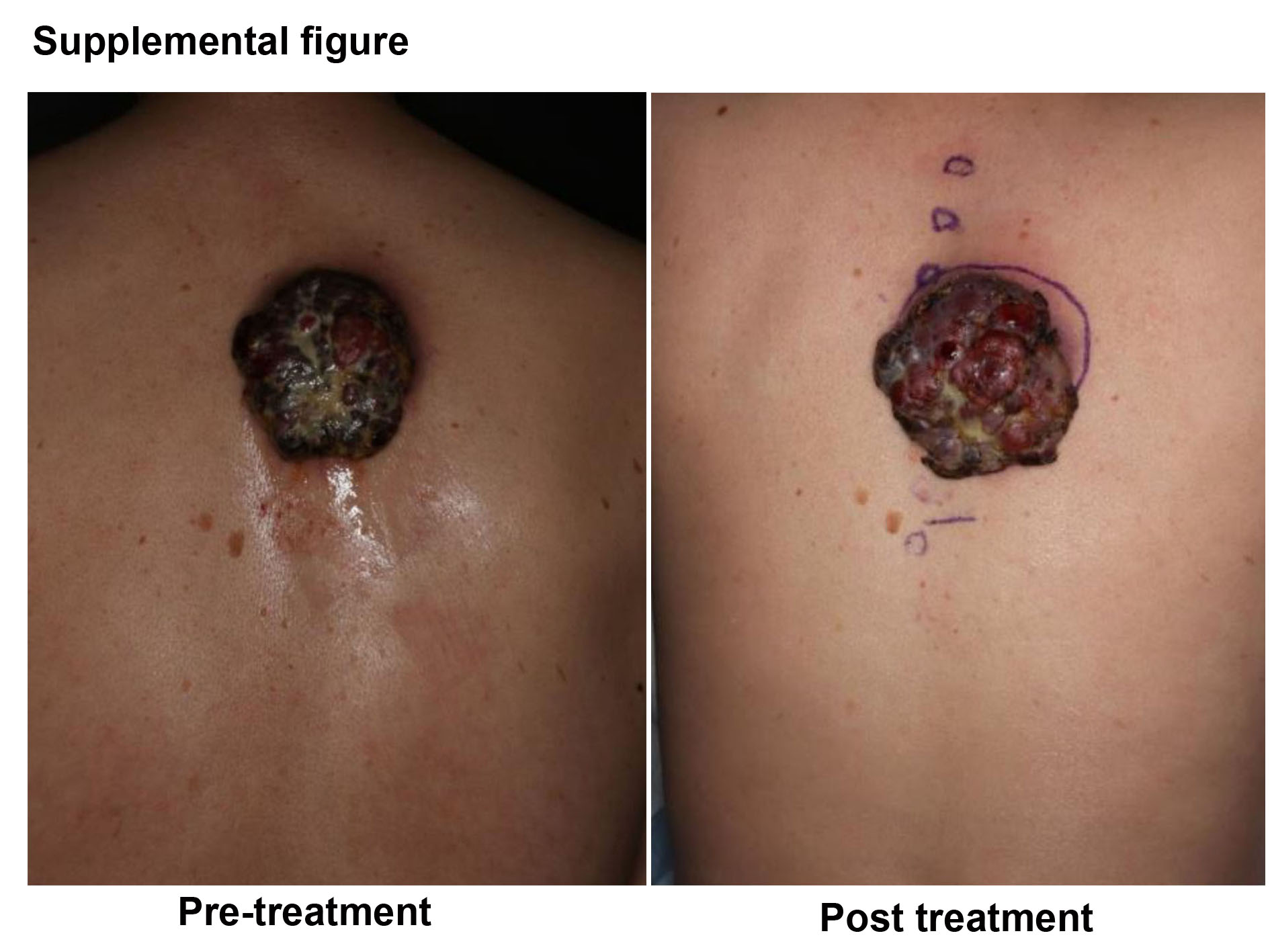

Supplement: Supplemental Figure 1 — Primary tumor on the back before and after the administration of nivolumab plus ipilimumab. [file Image_1.JPEG]
